# Mesenchymal Stem Cell-Derived Exosomes Attenuate Hepatic Steatosis and Insulin Resistance in Diet-Induced Obese Mice by Activating the FGF21-Adiponectin Axis

**DOI:** 10.3390/ijms251910447

**Published:** 2024-09-27

**Authors:** Bobae Kim, Rwubuzizi Ronaldo, Beet-Na Kweon, Solhee Yoon, Yein Park, Jea-Hyun Baek, Jung Min Lee, Chang-Kee Hyun

**Affiliations:** School of Life Science, Handong Global University, Pohang 37554, Gyungbuk, Republic of Korea

**Keywords:** exosome, insulin resistance, hepatic steatosis, FGF21, adiponectin, SIRT1

## Abstract

Exosomes derived from mesenchymal stem cells have shown promise in treating metabolic disorders, yet their specific mechanisms remain largely unclear. This study investigates the protective effects of exosomes from human umbilical cord Wharton’s jelly mesenchymal stem cells (hWJMSCs) against adiposity and insulin resistance in high-fat diet (HFD)-induced obese mice. HFD-fed mice treated with hWJMSC-derived exosomes demonstrated improved gut barrier integrity, which restored immune balance in the liver and adipose tissues by reducing macrophage infiltration and pro-inflammatory cytokine expression. Furthermore, these exosomes normalized lipid metabolism including lipid oxidation and lipogenesis, which alleviate lipotoxicity-induced endoplasmic reticulum (ER) stress, thereby decreasing fat accumulation and chronic tissue inflammation in hepatic and adipose tissues. Notably, hWJMSC-derived exosomes also promoted browning and thermogenic capacity of adipose tissues, which was linked to reduced fibroblast growth factor 21 (FGF21) resistance and increased adiponectin production. This process activated the AMPK-SIRT1-PGC-1α pathway, highlighting the role of the FGF21–adiponectin axis. Our findings elucidate the molecular mechanisms through which hWJMSC-derived exosomes counteract HFD-induced metabolic dysfunctions, supporting their potential as therapeutic agents for metabolic disorders.

## 1. Introduction

Exosomes are small membrane vesicles that play a crucial role in intercellular communication by transferring proteins, lipids, and nucleic acids between cells, and the effective transfer of cellular elements via exosomes provides valuable insights for their practical application in the development of therapies based on exosomes [1]. In the field of exosome therapy, the prevalence of research on mesenchymal stem cell (MSC)-derived exosomes in comparison to exosomes derived from other cell types can be attributed to their unique combination of regenerative, immunomodulatory, and versatile intercellular communication properties, making them promising candidates for the development of innovative therapeutic strategies. Recent studies have shown that MSC-derived exosomes have therapeutic potential for chronic inflammatory metabolic diseases, such as type 2 diabetes mellitus (T2DM), obesity, and metabolic dysfunction-associated steatotic liver disease (MASLD; formerly known as nonalcoholic fatty liver disease [NAFLD]). The molecular mechanisms underlying the therapeutic effects are complex and multifaceted, including anti-inflammatory immune modulation, enhancement of insulin signaling and energy metabolism, modulation of adipose tissue function. MSC-derived exosomes may transfer anti-inflammatory factors, including miRNAs and cytokines, which positively regulate insulin signaling pathways in target cells, thereby reducing chronic inflammation and improving glucose metabolism, and also modulate adipogenesis and lipid metabolism in adipocytes [2,3]. They may also carry antioxidants and immunomodulatory molecules that reduce oxidative stress in hepatocytes and hepatic inflammation, contributing to the improvement of MASLD [4,5,6].

Exosomes derived from various types of mesenchymal stem cell (MSC) types, including human umbilical cord mesenchymal stem cells (hucMSCs or hWJMSCs), bone marrow mesenchymal stem cells (BMMSCs), and adipose-derived stem cells (ADSCs), have been examined for their efficacy as therapeutic agents in treating chronic inflammatory metabolic diseases [2,7]. Recent research has highlighted the potential of hWJMSC-derived exosomes, noting their lower immunogenicity and higher biocompatibility. The exosomes activate insulin signaling and glucose metabolism in HFD-fed rats, leading to improved glucose uptake and glycolysis in skeletal muscle, as well as enhanced glycogen synthesis in the liver [8]. They also increase insulin sensitivity and adiponectin production in insulin-resistant human adipocytes [9]. Another study demonstrated that the use of hWJMSC-derived exosomes may restore the structural integrity of islets, enhance insulin sensitivity by promoting glucose uptake via GLUT1–4, and reduce insulin resistance in rats with T2DM [10]. Furthermore, it has been reported that hWJMSC-derived exosomes diminished hepatic fat accumulation in oleic–palmitic acid-treated hepatocytes and HFD-induced MASLD mice through the promotion of fatty acid oxidation and the inhibition of fatty acid synthesis [11]. Some studies have reported on the therapeutic mechanisms of action of exosomal microRNAs (miRs) derived from hWJMSCs on metabolic disorders [3]. For instance, it has been shown that hWJMSC-derived exosomal miR-17-3p ameliorates inflammatory reaction of diabetic retinopathy mice via targeting STAT1 [12], miR-24-3p targets Keap-1 to limit hepatic lipid accumulation, ROS production and inflammation [13], and miR-627-5p improves glucose and lipid metabolism and alleviate liver damage by repressing fat mass and obesity-associated (FTO) gene expression, thereby ameliorating MASLD progression [14]. In this way, research on the therapeutic effects of hWJMSC-derived exosomes has increased recently; however, the reported results are diverse and scattered, highlighting the need for further investigation to achieve a comprehensive understanding of their therapeutic mechanisms in chronic inflammatory metabolic diseases.

In this study, we investigated the mechanisms by which hWJMSC-derived exosomes exert protective effects against HFD-induced metabolic dysregulation in mice. Specifically, we focused on how these exosomes contribute to reduced adiposity and hepatic steatosis, enhanced insulin sensitivity, and improved lipid metabolism. We examined the positive effects of exosome treatment in detail, including improvements in gut barrier integrity, which helps prevent metabolic endotoxemia, and the restoration of immune balance, which is essential for reducing chronic inflammation. Additionally, we observed beneficial alterations in lipid metabolism, including enhanced lipid oxidation and reduced lipotoxicity, as well as a significant reduction in ER stress in both the liver and adipose tissues. We also noted the enhancement of browning and thermogenic capacity in adipose tissue, which contributes to increased energy expenditure. Our findings reveal that adiponectin and FGF21, which are key regulators of metabolic homeostasis, play crucial roles in mediating these beneficial effects through activating the SIRT1/PGC-1α pathway. Given the significant role of inter-organ metabolic crosstalk in maintaining energy homeostasis, our results provide valuable insights into the comprehensive mechanisms underlying the multiple protective effects of hWJMSC-derived exosomes against chronic inflammatory metabolic diseases.

## 2. Results

### 2.1. hWJMSC-Derived Exosomes Attenuate Diet-Induced Adiposity and Improve Insulin Sensitivity in HFD-Fed Obese Mice

**Figure 1:**
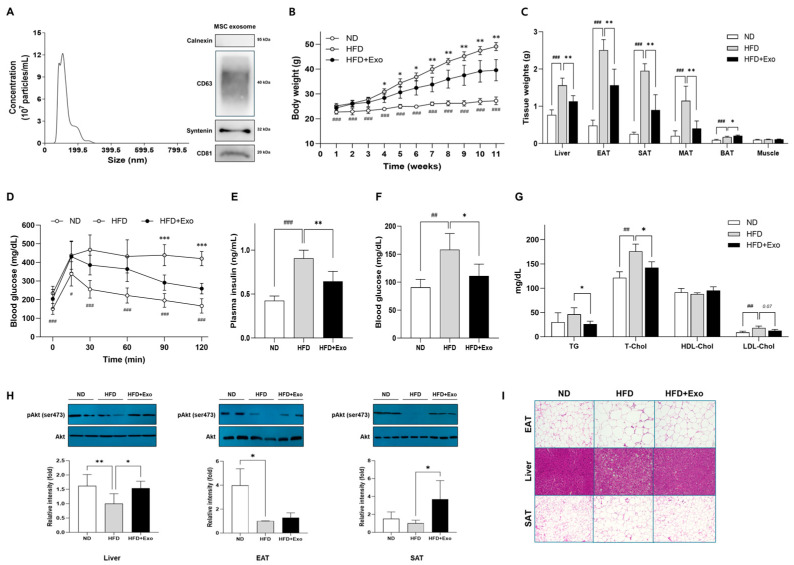
hWJMSC-derived exosomes reduce diet-induced adiposity and improve insulin sensitivity in HFD-fed mice. (**A**) Characterization of hWJMSC-derived exosomes by size analysis using nanoparticle tracking analysis (NTA) and Western blot analysis (Figure S1) of exosomal markers Calnexin, CD63, Syntenin, and CD81. (**B**) Body weight changes during 11 weeks of HFD feeding with exosome treatment (*n* = 9). (**C**) Tissue weights after 11 weeks of exosome treatment (*n* = 9). (**D**) Intraperitoneal glucose tolerance test in mice at 10 weeks of exosome treatment (*n* = 9). (**E**) Plasma concentration of insulin after 11 weeks of exosome treatment quantified by ELISA (*n* = 6). (**F**,**G**) Blood levels of glucose, TG, total cholesterol, and HDL and LDL cholesterol after 11 weeks of exosome treatment. (**H**) Akt phosphorylation levels in the liver, EAT, and SAT. (**I**) Representative images (×200) of H&E stained sections of the liver, EAT, and SAT. Data are presented as mean ± SD. ^#^ *p* < 0.05, ^##^ *p* < 0.01, and ^###^ *p* < 0.001 for ND vs. HFD, * *p* < 0.05, ** *p* < 0.01, and *** *p* < 0.001 for HFD vs. HFD+Exo. ND: normal chow diet-fed; HFD: high-fat diet-fed; HFD+Exo: exosome-treated HFD-fed group. EAT: epididymal adipose tissue; SAT: subcutaneous adipose tissue; MAT: mesenteric adipose tissue; BAT: brown adipose tissue; TG: triglyceride; T-chol: total cholesterol; HDL: high-density lipoprotein; LDL: low-density lipoprotein.

The mean diameter of extracellular vesicles isolated from the culture supernatant of hWJMSC, as measured by NTA was 124.7 ± 2.2 nm, and immunoblotting analysis confirmed that the vesicles were positive for exosome markers, including CD63, CD81, and syntenin, and negative for Calnexin (Figure 1A). The administration of hWJMSC-derived exosomes through tail intravenous injections to HFD-fed mice for 11 weeks led to reversed weight gain compared to HFD-fed control mice (Figure 1B). The HFD-induced increases in the weights of the liver and white adipose tissues (WATs) including epididymal, mesenteric and subcutaneous adipose tissues (EAT, MAT, and SAT, respectively) were significantly reversed by the treatment of exosomes, whereas interestingly, the weight of brown adipose tissue (BAT) significantly increased in exosome-treated HFD-fed mice compared to untreated HFD-fed control mice (Figure 1C).

Importantly, exosome-treated HFD-fed mice also demonstrated significant improvement in glucose tolerance compared to untreated HFD-fed control mice (Figure 1D). Together with this, the plasma levels of insulin and fasting glucose were significantly reduced (Figure 1E,F) and Akt phosphorylation in the liver and WATs was significantly increased (Figure 1H) in exosome-treated mice compared to controls, indicating that exosome treatment enhanced insulin sensitivity. Moreover, plasma levels of triglyceride (TG), total cholesterol (T-Chol) and low-density lipoprotein cholesterol (LDL-Chol) were significantly reduced in the exosome-treated group (Figure 1G). In addition, exosome-treated HFD-fed mice also showed a significant decrease in ectopic fat deposition in liver and WATs (EAT, SAT), which was confirmed by histological observation of the liver, EAT, and SAT compared to untreated HFD-fed mice (Figure 1I).

### 2.2. hWJMSC-Derived Exosomes Restore Immune Homeostasis in the Liver and Adipose Tissues of HFD-Fed Obese Mice

**Figure 2:**
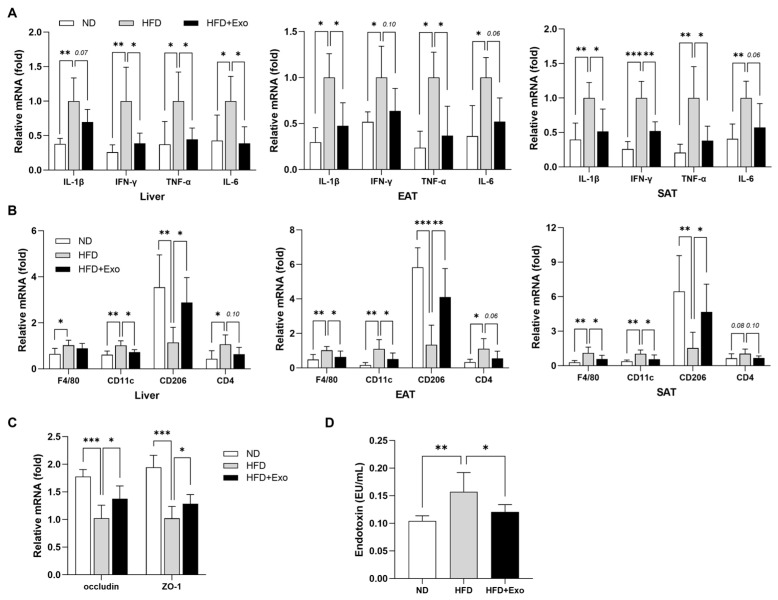
hWJMSC-derived exosomes restore immune homeostasis in the liver and adipose tissues of HFD-fed obese mice. Changes in mRNA expression of (**A**) pro-inflammatory cytokines and (**B**) immune cell markers in the liver, EAT, and SAT, and (**C**) tight junction proteins in the colon. The relative mRNA levels were analyzed by real-time PCR for the indicated genes and normalized to the expression of GAPDH or Arbp gene. (**D**) Plasma levels of LPS measured by the LAL assay. Data are presented as mean ± SD for 6~8 mice in each group. * *p* < 0.05, ** *p* < 0.01, and *** *p* < 0.001. ND: normal chow diet-fed; HFD: high-fat diet-fed; HFD+Exo: exosome-treated HFD-fed group; EAT: epididymal adipose tissue; SAT: subcutaneous adipose tissue.

Chronic tissue inflammation plays a significant role in the development of obesity-induced adiposity and insulin resistance [15]. To examine the potential of exosome in the improvement of chronic inflammation induced by HFD, we accessed the mRNA expression of pro-inflammatory cytokines and immune cell markers in the liver, EAT, and SAT. HFD-fed mice treated with exosomes showed a significant reduction in the expression of IL-1β, IFN-γ, TNF-α, and IL-6 in all those tissues compared to untreated control mice (Figure 2A). There was also a significant decrease in the expressions of the pan-macrophage marker F4/80 and the pro-inflammatory M1 macrophage marker CD11c, while the expression of an anti-inflammatory M2 macrophage marker, CD206, was significantly upregulated in exosome-treated mice (Figure 2B). Furthermore, exosome-treated mice exhibited a noteworthy upregulation in the expression of tight junction-associated proteins, including occludin and ZO-1, in the colon (Figure 2C). This increased expression of tight junction-associated proteins coincided with a significant decline in plasma LPS levels in the exosome-treated mice compared to controls (Figure 2D). Together, these results suggest that the protective effect of exosome treatment against HFD-induced chronic inflammation and metabolic abnormalities was, at least in part, linked to enhancement in gut barrier integrity.

### 2.3. hWJMSC-Derived Exosomes Restore Lipid Metabolism and Attenuate ER Stress in the Liver and Adipose Tissues of HFD-Fed Obese Mice

**Figure 3:**
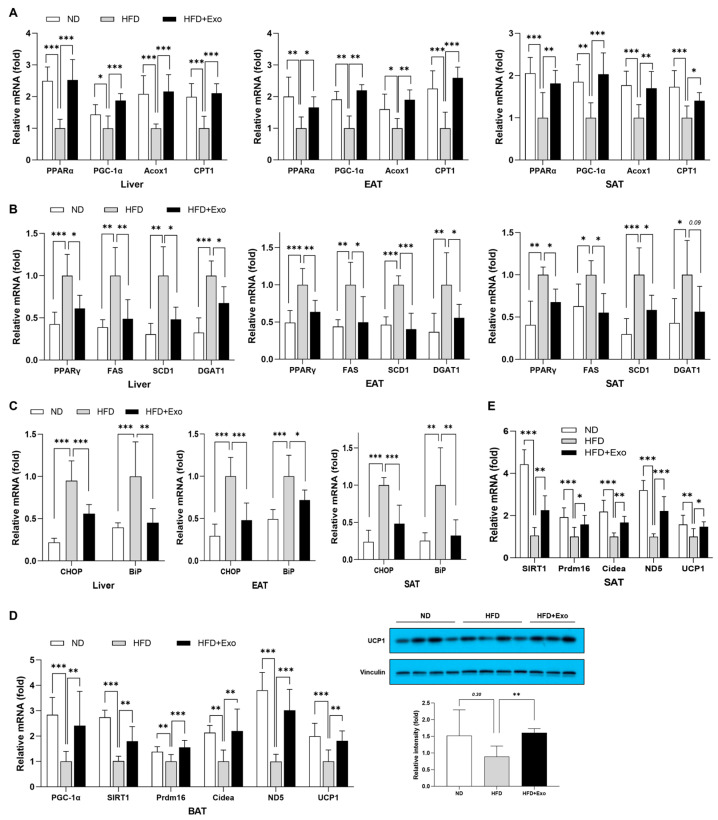
hWJMSC-derived exosomes restore lipid metabolism, attenuate ER stress, and promote SAT browning and BAT thermogenesis in HFD-fed obese mice. Changes in mRNA expression of (**A**) genes involved in lipid oxidation, (**B**) de novo lipogenic genes, and (**C**) ER stress-related proteins in the liver, EAT, and SAT. Changes in mRNA expression of browning and thermogenesis in BAT (**D**) and SAT (**E**). The relative mRNA levels were analyzed by real-time PCR for the indicated genes and normalized to the expression of GAPDH or Arbp gene. Data are presented as mean ± SD for 6~8 mice in each group. * *p* < 0.05, ** *p* < 0.01, and *** *p* < 0.001. ND: normal chow diet-fed; HFD: high-fat diet-fed; HFD+Exo: exosome-treated HFD-fed group; EAT: epididymal adipose tissue; SAT: subcutaneous adipose tissue.

In an obese condition, increased circulating fatty acids due to adipose tissue dysfunction causes hepatic steatosis and lipotoxicity along with increased ER stress, consequently leading to hepatocyte damage and inflammation [16,17]. To further explore the molecular mechanisms by which hWJMSC-derived exosomes attenuated HFD-induced adiposity and subsequent inflammation in the liver and WATs, we assessed the changes in the expression of genes related to lipid oxidation, lipogenesis, and ER stress in the liver and adipose tissues. We observed that the expression of genes involved in lipid oxidation such as PPARα and PGC-1α, and their target genes Acox1 and CPT1 showed a significant increase, whereas the expression of de novo lipogenic genes—PPARγ, SREBP1c, FAS, SCD1, and DGAT1—was significantly suppressed in the liver, EAT, and SAT of exosome-treated mice compared to non-treated controls (Figure 3A,B). In addition, exosome-treated mice showed a significant decrease in ER stress-related proteins such as CHOP and BiP in the liver, EAT, and SAT, suggesting an improvement in ER stress-induced inflammation in each tissue of exosome-treated mice (Figure 3C). Taken together, these results suggest that exosome treatment to HFD-fed mice restored lipid metabolism including lipid oxidation and lipogenesis, thereby reducing lipotoxicity-induced ER stress and alleviating hepatic and adipose fat accumulation as well as chronic tissue inflammation.

### 2.4. hWJMSC-Derived Exosomes Promote WAT Browning and BAT Thermogenesis in HFD-Fed Obese Mice

There has been growing acknowledgement that targeting BAT thermogenesis along with WAT browning could potentially be a novel therapeutic approach to combat obesity [18]. To assess whether the amelioration of HFD-induced adiposity and insulin resistance in mice treated with hWJMSC-derived exosomes resulted from enhanced energy expenditure through non-shivering thermogenesis via BAT activation and WAT browning, we examined alterations in the expression of related genes. The mRNA levels of thermogenic genes such as ND5, Prdm16, Cidea, and UCP1 were found to be significantly elevated in both BAT and SAT of exosome-treated mice compared to untreated HFD-fed controls (Figure 3D,E). This elevation correlated with an upregulation of their master regulators SIRT1 and PGC-1α in those tissues (Figure 3A,D,E), indicating that hWJMSC-derived exosomes have a capacity to promote SAT browning and the thermogenic capacity of SAT and BAT.

### 2.5. hWJMSC-Derived Exosomes Activate the FGF21–Adiponectin Axis in the Liver and Adipose Tissues of HFD-Fed Obese Mice

**Figure 4:**
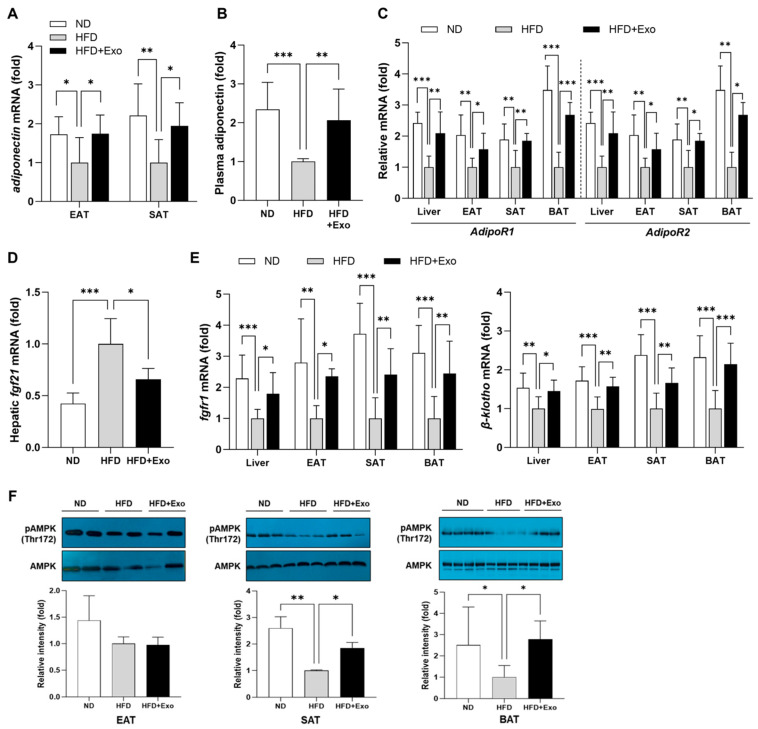
hWJMSC-derived exosomes activate the FGF21–adiponectin axis in the liver and adipose tissues of HFD-fed obese mice. Changes in (**A**) adiponectin mRNA expression in EAT and SAT and (**B**) plasma level of adiponectin. mRNA expression of (**C**) adiponectin receptors, (**D**) hepatic FGF21, and (**E**) FGF21 receptor (FGFR1) and co-receptor β-klotho in each tissue. The relative mRNA levels were analyzed by real-time PCR for the indicated genes and normalized to the expression of GAPDH or Arbp gene. (**F**) AMPK phosphorylation level in EAT, SAT, and BAT. Data are presented as mean ± SD for 6~8 mice in each group. * *p* < 0.05, ** *p* < 0.01, and *** *p* < 0.001. ND: normal chow diet-fed; HFD: high-fat diet-fed; HFD+Exo: exosome-treated HFD-fed group; EAT: epididymal adipose tissue; SAT: subcutaneous adipose tissue; BAT: brown adipose tissue.

To access the beneficial impact of exosome treatment on HFD-induced metabolic dysfunctions, we evaluated the production of adiponectin and the modulation of intestinal permeability. Adiponectin, an anti-inflammatory adipokine, is known for its beneficial effect on the metabolism and cardiovascular system and protecting against metabolic disorders [19]. The exacerbation of intestinal permeability, leading to an elevated level of circulating LPS, is a key factor contributing to chronic inflammation under HFD-fed conditions [20]. Our findings in this study revealed a significant increase in the expression of adiponectin in WATs of exosome-treated HFD-fed mice compared to those in untreated HFD-fed controls (Figure 4A). Changes in plasma adiponectin level also showed the same trend (Figure 4B). Consistently with this, the expression levels of adiponectin receptors, AdipoR1 and AdipoR2 in the liver, WATs, and BAT were also significantly higher in the exosome-treated group than the control group (Figure 4C).

Additionally, considering that impaired adaptive thermogenesis is related to an elevated susceptibility to hepatic steatosis, we examined alterations in the expression of a crucial regulator of the thermogenic program, FGF21, in the liver. In HFD-fed mice, there was a notable increase in hepatic mRNA expression of FGF21 compared to those of ND-fed mice (Figure 4D), indicating that HFD feeding induced a state of FGF21 resistance leading to compensatory FGF21 overproduction. However, the hepatic mRNA expression of FGF21 was significantly decreased in exosome-treated HFD-fed mice compared to their HFD-fed controls. Also, while HFD mice showed significantly lower mRNA expression of FGFR1 and β-klotho in EAT, SAT, and BAT than ND mice, it was significantly reversed by exosome treatment (Figure 4E). Notably, this was commensurate with a significant enhancement of AMPK phosphorylation in adipose tissues of exosome-treated mice (Figure 4F). Together with the data related to adiponectin, these data suggested that the improvement of metabolic dysfunctions in exosome-treated mice was associated with an attenuated FGF21 resistance and a consequent activation of the FGF21–adiponectin axis.

## 3. Discussion

As cell-based therapeutic approaches advance, MSCs have found extensive applications in treating chronic inflammatory metabolic diseases such as diabetes, obesity, and MASLD [21,22]. Among the various sources of MSCs, human umbilical cord stands out as a preferred candidate for cell-based therapies due to its non-invasive acquisition, absence of ethical concerns, and lower immunogenicity, distinguishing it from other sources [23,24]. Furthermore, mounting evidence suggests that hWJMSC-derived exosomes represent innovative cell-free carriers characterized by minimal immunogenicity, which have the capacity to attenuate harmful immune responses in inflamed tissues [4,25,26]. Hence, it is essential to further explore and comprehend the underlying mechanisms by which hWJMSC-derived exosomes play a role in the treatment of metabolic disorders through modulating immune responses and consequently improving both glucose and lipid metabolism.

Our investigation revealed the protective efficacy of hWJMSC-derived exosomes against metabolic impairments induced by HFD in mice, and we delved into the exploration of the molecular mechanisms responsible for these effects. The treatment of hWJMSC-derived exosomes mitigated the HFD-induced increase in body weight, primarily ascribed to a significant decrease in the liver weight and mass of white adipose tissues compared to untreated control mice. Simultaneously, alongside the reduction in body weight gain, exosome treatment led to an enhancement in insulin sensitivity, as evidenced by improved glucose tolerance, reduced plasma insulin levels, and promoted insulin signaling in the liver and WATs. Chronic inflammation linked to obesity is recognized as a key factor contributing to diminished insulin sensitivity [27]. We noted in our study that the treatment of exosomes led to a decrease in the expression of pro-inflammatory cytokines such as TNF-α, IL-6, IL-1β, and IFN-γ in the liver and WATs, and a modulation of immune cell marker expressions. This was accompanied by a recovery of the HFD-induced decrease in tight junction protein expression and increase in plasma endotoxin level, indicating that exosome treatment could reverse the intestinal barrier dysfunction and thus improve endotoxemia-induced inflammation in the metabolic tissues [15,20]. These results suggest that the improvement in insulin sensitivity through exosome treatment was associated with the attenuation of HFD-induced chronic inflammation in peripheral tissues.

However, these findings raised a question of whether the effects of exosomes resulted solely from a single action of alleviating chronic inflammation, without the involvement of any other actions. According to recent in vitro studies conducted with cell models, hWJMSC-derived exosomes were found to have direct effects, such as enhancing insulin sensitivity in insulin-resistant human adipocytes [9] and promoting fatty acid oxidation while simultaneously inhibiting fat synthesis in oleic–palmitic acid-treated hepatic cells [11]. From these, it can be inferred that the actions of hWJMSC-derived exosomes are not limited to alleviating chronic inflammation. Thus, we directed our focus to the comprehensive analysis of lipid metabolic pathways in the liver and adipose tissues. Data obtained in this study reveal that exosome treatment significantly attenuated hepatic steatosis and adipocyte hypertrophy.

MASLD is a prevalent chronic metabolic liver disorder characterized by the accumulation of fat in the liver [28]. In our research, exosome treatment is shown to result in a notable reduction in HFD-induced hepatomegaly and hepatic steatosis, which was supported by an increased expression of lipid oxidative genes and a decreased expression of lipogenic genes in the liver. We also observed that exosome treatment also improved the plasma lipid profile. Consistently with reports that cholesterol content increases due to increased hepatic fat synthesis [29], the plasma levels of TG, total cholesterol, and LDL cholesterol were increased by HFD feeding, and were significantly reduced by exosome treatment. These observations suggest that exosomes enhance fat oxidation and simultaneously suppress de novo fat synthesis, ultimately contributing to its protective impact against the features of MASLD and dyslipidemia. These results are consistent with the findings of Yang et al., who treated hWJMSC-derived exosomes to HFD-induced MASLD mice and observed reductions in hepatic fat accumulation and the levels of plasma TG and cholesterol [11]. Their study concluded that calcium/calmodulin-dependent protein kinase 1 (CAMKK1) transferred by exosomes mediates lipid homoeostasis regulation via AMPK-mediated signaling in hepatocytes, leading to a protection against HFD-induced MASLD. However, in this study, we additionally observed that exosomes reduced lipotoxicity-induced endoplasmic reticulum (ER) stress and mitochondrial dysfunction in hepatocytes, which indicates that the regulatory action of exosomes on lipid metabolism need to be more comprehensively analyzed, including crosstalk with adipose tissue. Hepatic lipotoxicity manifests when there is a substantial influx of FFAs from the peripheral tissues, mainly the adipose tissue, leading to the synthesis and accumulation of TG and toxic levels of fatty acids, free cholesterol, and other lipid metabolites. These factors contribute to mitochondrial dysfunction, oxidative stress, and ER stress, triggering the activation of the unfolded protein response (UPR), which collectively contribute to hepatic inflammation [4,30]. The intricate interplay among distant organs plays a crucial role in regulating whole-body energy metabolism, and this coordination is mainly achieved by various hormones released from each organ. Notably, among them, FGF21 and adiponectin have gained considerable attention due to their diverse protective effects against a range of metabolic disorders [31]. The lipotoxicity-induced ER stress and mitochondrial dysfunction also can be reduced by the actions of this FGF21–adiponectin axis [32,33].

Adipocyte hypertrophy refers to an excessive accumulation of lipids in adipocytes beyond the capacity, leading to various metabolic disruptions such as ectopic fat accumulation, chronic inflammation, and insulin resistance [34,35]. We observed in this study that, in addition to reducing hepatic adiposity, exosome treatment also reversed the HFD-induced weight gain and adiposity, accompanied by a significant reduction in adipocyte hypertrophy in WATs of mice subjected to exosome treatment. Moreover, the upregulation of genes involved in SAT browning and BAT thermogenesis was also significant in exosome-treated mice. This observation of the present study, coupled with an upregulation of lipid oxidative genes and a downregulation of lipogenic genes in the exosome-treated conditions, indicates that hWJMSC-derived exosomes have a capacity to modulate lipid metabolism in all metabolic tissues including the liver, WATs, and BAT and this modulation results in suppressed HFD-induced adiposity and consequently enhanced metabolic control.

To explore the molecular mechanism underlying the suppressing effect of hWJMSC-derived exosomes against HFD-induced metabolic disturbance, taking into account the crucial interplay between the liver and adipose tissues in maintaining energy homeostasis, we postulated that hWJMSC-derived exosomes could potentially alleviate the disruption of inter-organ metabolic homeostasis. There is substantial evidence indicating a strong correlation between hepatic fat accumulation and an irregularity in lipid metabolic processes such as fatty acid uptake, de novo lipogenesis, fatty acid oxidation, and lipid export [36], which is attributed to the dysregulation of mediators involved in inter-organ metabolic communication, including FGF21 and adiponectin. Additionally, their pivotal regulator SIRT1, a NAD^+^-dependent deacetylase, plays crucial roles in the control of lipid metabolism [37,38,39]. SIRT1 enhances hepatic fatty acid oxidation via the PPARα/PGC-1α pathway [40] and induces a brown-like phenotype in SAT through PPARγ deacetylation [41]. It also stimulates BAT thermogenesis by PGC-1α-mediated upregulation of mitochondrial genes [42]. Furthermore, SIRT1 serves a protective function against hepatic inflammation by inhibiting NF-κB-mediated induction of pro-inflammatory cytokines, while, in chronic inflammatory conditions, SIRT1 regulation becomes disrupted [43].

The induction of SAT browning and BAT thermogenesis, recognized as significant contributors to energy expenditure, exert an influence on systemic lipid balance as well, and various secreted factors such as FGF21 and adiponectin have been identified as activators of these metabolic processes [44,45,46]. In order to exert its effects on target tissues, FGF21 necessitates binding to FGF receptors, primarily FGFR1, and the essential coreceptor β-klotho [47]. In WATs, FGF21 stimulates glucose uptake, promotes mitochondrial function, and enhances the effect of PPAR-γ, which are achieved through AMPK-SIRT1-PGC-1α signaling-dependent mechanisms [48]. Furthermore, FGF21 has the capability to promote the thermogenic activity of BAT and induce the browning of WAT by acting on PGC-1α to control the expression of thermogenic genes including UCP1, leading to an enhancement of energy expenditure and glucose tolerance [49]. FGF21 triggers the production of adiponectin in adipocytes through its binding to FGFR1 and β-klotho, and consequently, adiponectin acts as a crucial mediator for FGF21’s function, regulating glucose and lipid metabolism as well as insulin sensitivity [50]. Notably, in mice lacking adiponectin, the beneficial effects of FGF21, such as alleviating obesity-associated insulin resistance, hyperglycemia, hyperlipidemia, and hepatic steatosis, are diminished [51]. However, under the condition of obesity-induced metabolic disturbance, circulating FGF21 levels increase while adiponectin levels decrease in both animals and humans. This discrepancy may be attributed to FGF21 resistance and implies that the dysfunctional FGF21–adiponectin axis contributes to the pathogenesis of obesity-related metabolic syndrome [51]. Interestingly, exercise appears to prevent the HFD-induced reduction in FGF21 action of promoting adiponectin secretion, and this protective effect against HFD-induced FGF21 resistance is associated with the increased expression of FGFR1 and β-klotho in adipose tissues [52,53]. In this study, we observed that the dysregulated expression of hepatic FGF21 and its receptors in the liver, WATs, and BAT caused by HFD was recovered by the treatment of exosome, which was commensurate with a restoration of HFD-induced decrease in adiponectin and its receptors. This suggests that hWJMSC-derived exosomes could reverse HFD-induced FGF21 resistance and promote the activation of the FGF21–adiponectin axis.

The results from our study also reveal that hWJMSC-derived exosomes restore hepatic lipid metabolism by activating SIRT1-mediated metabolic pathways, including fatty acid oxidation, SAT browning, and BAT thermogenesis. We observed a significant upregulation not only in the expression of genes associated with SAT browning and BAT thermogenesis but also in SIRT1 expression in the liver, SAT, and BAT in mice treated with exosomes. This enhancement was accompanied by the increased expression of genes related to fatty acid oxidation in the liver and WATs. Collectively, these findings suggest that hWJMSC-derived exosomes may mitigate HFD-induced metabolic dysfunction by reinforcing SIRT1-mediated metabolic processes, which consequently contributes to the protection against hepatic steatosis and insulin resistance. Accumulated evidence indicates that the decreased expression of adiponectin is a prominent characteristic of chronic inflammation in metabolic disorders [54]. Under these conditions of chronic inflammation, there is a decline in cellular NAD^+^ levels in metabolic organs, leading to reduced SIRT1 activity. Adiponectin, however, counters this effect by activating AMPK and subsequently inducing SIRT1/PGC-1α-mediated mitochondrial functions, which include fatty acid oxidation, SAT browning, and BAT thermogenesis [55,56]. Our data, illustrating an increase in the expression of adiponectin, AdipoR1, and AdipoR2 in the liver and adipose tissues, along with heightened AMPK activation in these tissues of mice treated with exosomes, in conjunction with the elevated expression of SIRT1, suggest that hWJMSC-derived exosomes may reverse the HFD-induced disruption of adiponectin-AMPK-SIRT1-mediated metabolic regulation.

**Figure 5:**
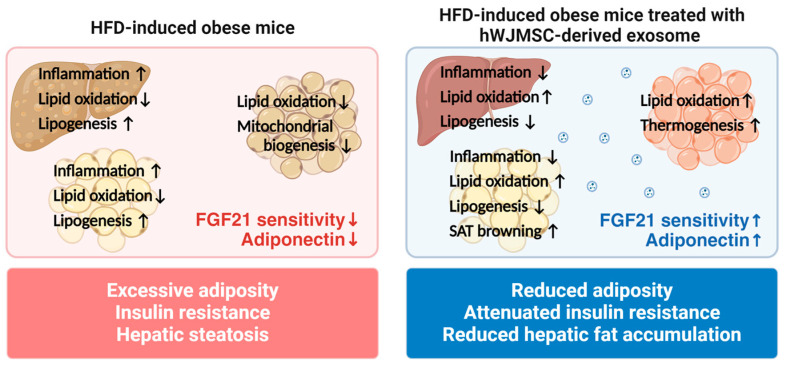
A summary of possible mechanisms that explain how hWJMSC-derived exosomes protect against metabolic dysregulation in HFD-fed obese mice.

In conclusion, this study illustrates that hWJMSC-derived exosomes hold potential as a therapeutic candidate for ameliorating HFD-induced adiposity, insulin resistance, and hepatic steatosis. Our results allow us to propose a model, shown in Figure 5, that elucidates a possible mechanism for the improvement of metabolic disorders mediated by hWJMSC-derived exosomes. As depicted in the diagram, the beneficial effects of hWJMSC-derived exosomes arise from suppression of chronic inflammation and restoration of perturbed glucose and lipid metabolic homeostasis. Notably, the latter is contributed by the activation of the FGF21–adiponectin axis, which subsequently stimulates the AMPK/SIRT1/PGC-1α pathway in the liver and adipose tissues. Our findings provide a molecular mechanism how hWJMSC-derived exosomes mitigate the dysregulation of glucose and lipid homeostasis. This conclusively positions hWJMSC-derived exosomes as a promising therapeutic agent for the prevention and treatment of metabolic disorders.

## 4. Materials and Methods

### 4.1. Isolation and Characterizations of Exosomes

hWJMSCs were cultured in low-glucose DMEM (Welgene, Daegu, Republic of Korea) supplemented with fetal bovine serum (Corning Inc., Corning, NY, USA) and penicillin/streptomycin (Welgene) at 37 °C in 5% CO_2_ incubator. MSC surface markers (CD73, CD90, and CD105) were used to identify hWJMSCs by conventional flow cytometry (Supplementary Figure S2). When the confluency reached 90%, the culture medium was changed to low-glucose serum-free DMEM (Welgene) and incubated. After 24 h, medium was harvested, and exosomes were isolated through a tangential flow filtration (TFF) system. At the last step of isolation, particles larger than exosomes were removed by sterile filtration. The size of the exosome particles was analyzed by nanoparticle tracking analysis (NTA) using NanoSight NS300 (Malvern Panalytical, Malvern, UK). The expression of calnexin, CD63, CD81, and syntenin were detected by Western blotting. Exosome samples were diluted in PBS for further experiments. hWJMSCs, which were isolated from the Wharton′s jelly of human umbilical cord tissue, were kindly gifted from Samsung Medical Center (Seoul, Republic of Korea). The study involving human participants were reviewed and approved by the Institutional Review Board of Samsung Medical Center (Approval no. 2015-10-025), and all samples were obtained with informed consent.

### 4.2. Animal Experiments

Six-week-old C57BL/6J male mice purchased from Central Lab. Animal Inc. (Seoul, Republic of Korea) were housed at 22 ± 1 °C and 45 ± 10% humidity, on a 12 h light/dark cycle. After 2 weeks of adaption, mice were divided into three experimental groups (*n* = 9 per group) each receiving different treatments: normal diet (ND)-fed control (ND), HFD-fed untreated control (HFD), and HFD-fed exosome-treated (HFD+Exo) groups. During 11 weeks of ND (10%kcal from fat, D12450J, Research Diets Inc., New Brunswick, NJ, USA) or HFD (60% kcal from fat, D12492, Research Diets Inc.) feeding, mice were intravenously administered via tail veil with 200 µL PBS (ND and HFD group) or hWJMSC-derived exosomes in 200 µL PBS (HFD+Exo group) at a dose of 5 × 10^9^ particles/mouse every other day. On the last day of the experiment, mice were sacrificed, and tissue samples were harvested as previously described [54]. Briefly, tissues of the liver, quadriceps skeletal muscle, subcutaneous adipose tissue (SAT), epididymal adipose tissue (EAT), mesenteric adipose tissue (MAT), and interscapular brown adipose tissue (BAT) were harvested, snap-frozen in liquid nitrogen, and stored at −70 °C until processed for RNA and protein analysis. To prepare blood plasma samples, fresh blood obtained via cardiac puncture was collected in a BD Microtainer PST™ tube (BD Scientific, Franklin Lakes, NJ, USA) and subsequently centrifuged at 15,000× *g* for 5 min, and the separated plasma was frozen at −80 °C until analysis. All animal experiments were performed in accordance with protocols approved by the Institutional Animal Care and Use Committee of Handong Global University (Permit number: IACUC20221123-17).

### 4.3. Blood Plasma Analyses

Plasma levels of triglyceride (TG), total cholesterol, high-density lipoprotein (HDL) cholesterol, and low-density lipoprotein (LDL) cholesterol were measured using an automated analyzer, Mindray BS-390 (Mindray Bio-Medical Electronics Co., Shenzhen, China). Plasma levels of lipopolysaccharide (LPS), insulin and adiponectin were measured using ToxinSensor™ Chromogenic LAL Endotoxin Assay Kit (GenScript, Piscataway, NJ, USA), a mouse insulin ELISA kit (ALPCO, Salem, NH, USA), and a mouse adiponectin/Acrp30 Quantikine ELISA kit (R&D systems, Minneapolis, MN, USA), respectively.

### 4.4. Glucose Tolerance Test

After 8 weeks of exosome treatment, mice were fasted for 16 h, with free access to water, prior to the test. Glucose was injected intraperitoneally at a concentration of 2 g/kg body weight, and the glucose levels in blood samples from tail bleeds were measured using GlucoDr auto AGM-4000 (Allmedicus, Anyang, Republic of Korea) at baseline and 15, 30, 60, 90, and 120 min after glucose injection.

### 4.5. Histological Analysis

Liver, EAT, and SAT samples fixed in 10% *v*/*v* formalin/PBS were embedded in paraffin, and then 5 μm thick microtome sections were prepared and stained with hematoxylin and eosin. Images were obtained under a microscope at a magnification of 200×.

### 4.6. Real-Time RT PCR

Total RNA was extracted using NucleoZOL (Macherey-Nagel, Düren, Germany) and complementary DNA was synthesized by reverse transcription of 2 μg of extracted RNA using the GoScript™ reverse transcription system (Promega, Madison, WI, USA) according to the manufacturer’s instructions. Quantitative real-time PCR was performed using GoTaq^®^ qPCR Master Mix (Promega) on a StepOnePlus™ Real-Time PCR system (Applied Biosystems, Foster City, CA, USA). Quantification of gene transcripts for adiponectin, adiponectin receptor 1 (AdipoR1), AdipoR2, acidic ribosomal phosphoprotein (Arbp), acyl-CoA oxidase 1 (ACOX1), binding of immunoglobulin protein (BiP), carnitine palmitoyltransferase 1 (CPT1), CCAAT/enhancer-binding protein homologous protein (CHOP), cell death-inducing DNA fragmentation factor α-like effector A (Cidea), cluster of differentiation 4 (CD4), CD11c, CD206, diacylglycerol acyltransferase 1 (DGAT1), F4/80, fatty acid synthase (FAS), fibroblast growth factor 21 (FGF21), fibroblast growth factor receptor 1 (FGFR1), forkhead box P3 (FOXP3), glyceraldehyde-3-phosphate dehydrogenase (GAPDH), interferon γ (IFN-γ), interleukin-1β (IL-1β), IL-6, β-klotho, NADH-ubiquinone oxidoreductase chain 5 (ND5), occludin, peroxisome proliferator-activated receptor α (PPARα), PPARγ, PPARγ coactivator-1α (PGC-1α), PR domain-containing 16 (Prdm16), sirtuin 1 (SIRT1), stearoyl-CoA desaturase 1 (SCD1), sterol-regulatory element binding protein 1c (SREBP1c), tumor necrosis factor α (TNF-α), uncoupling protein 1 (UCP1), ZO-1 was performed using gene-specific forward and reverse primers. Primer sequences are available in Supplementary Table S1. The relative expression levels of each gene were calculated using the ΔΔCt method and normalized to the expression of Arbp for adipose tissues and the colon and GAPDH for the liver tissue.

### 4.7. Western Blot Analysis

Western blot analysis was performed as described previously [57]. Antibodies against AMPK, phospho (Thr172) AMPK, adiponectin (Cell Signaling Technology, Beverly, MA, USA), calnexin, CD63, CD81 (Bioss Antibodies, Woburn, MA, USA), GAPDH (Cell Signaling Technology), occludin, and syntenin (Bioss Antibodies) were used as primary antibodies, and blots were incubated with the appropriate IgG-HRP-conjugated secondary antibody. Immunoblots were visualized by ECL, and densitometric analyses were performed using ImageJ software version 1.54g.

### 4.8. Statistical Analyses

All data were presented as means ± SD for 6–8 mice per each group. The statistical significance between groups was analyzed using GraphPad Prism software version 10.1.2 (GraphPad, San Diego, CA, USA), and statistical comparisons were carried out using a one-way or two-way analysis of variance (ANOVA) with Fisher’s LSD test or Dunnett’s multiple comparison test. *p* values < 0.05 (* *p* < 0.05, ** *p* < 0.01, *** *p* < 0.001) were considered as statistically significant.

## Data Availability

The original contributions presented in the study are included in the article and Supplementary Materials. Further inquiries can be directed to the corresponding author.

## Supplementary Materials

The supporting information can be downloaded at: https://www.mdpi.com/article/10.3390/ijms251910447/s1.
