# Supplementary material for: Mesenchymal Stem Cell-Derived Exosomes Attenuate Hepatic Steatosis and Insulin Resistance in Diet-Induced Obese Mice by Activating the FGF21-Adiponectin Axis
Source: Int J Mol Sci. 2024 Sep 27;25(19):10447. doi: 10.3390/ijms251910447 (PMC11476820; doi:10.3390/ijms251910447)
Supplement: Supplementary file 1 [file ijms-25-10447-s001.zip › ijms-3220901-supplementary.pdf]

Supplementary Material

Mesenchymal-stem cell-derived exosomes attenuate hepatic steatosis and insulin resistance in diet-induced obese mice by activating the FGF21-adiponectin axis

Bobae Kim, Rwubuzizi Ronaldo, Beet-Na Kweon, Solhee Yoon, Yein Park, Jae-Hyun Baek, Jung Min Lee, Chang-Kee Hyun*

School of Life Science, Handong Global University, Pohang, Gyungbuk, Republic of Korea

*** Correspondence:**
Chang-Kee Hyun
ckhyun@handong.edu

Supplementary Table S1. Primer sequences for real-time PCR

Gene		Primer sequence
Adiponectin	Forward	GCAGAGATGGCACTCCTGGA
	Reverse	CCTTCAGCTCCTGTCATTCC
AdipoR1	Forward	TGGCTGATAACGGGCCATC
	Reverse	GGCGTGGCTTTGTTTGTCC
AdipoR2	Forward	GGAGATTTGGAGCCCAGCTT
	Reverse	GGCCTTCCCACACCTTACAA
Arbp	Forward	TCACTGTGCCAGCTCAGAAC
	Reverse	AATTTCAATGGTGCCTCTGG
ACOX1	Forward	GTGCAGCTCAGAGTCTGTCCAA
	Reverse	TACTGCTGCGTCTGAAAATCCA
BiP	Forward	GCGTGTGTGTGAGACCAGAA
	Reverse	CAGTCGCTCAGCAGTCAGG
CPT1	Forward	TGAGTGGCGTCCTCTTTGG
	Reverse	TCAGCGAGTAGCGCATAGTCA
CHOP	Forward	GTCCAGCTGGGAGCTGGAAG
	Reverse	CTGACTGGAATCTGGAGAG
Cidea	Forward	TGCTCTTCTGTATCGCCCAGT
	Reverse	GCCGTGTTAAGGAATCTGCTG
CD4	Forward	CCAGACAGTGTTCCTGGCTT
	Reverse	TGCCTGGCGCTGTTGG
CD11c	Forward	ATGGAGCCTCAAGACAGGAC
	Reverse	GGATCTGGGATGCTGAAATC
CD206	Forward	GTGGAGTGATGGAACCCCAG
	Reverse	CTGTCCGCCCAGTATCCATC
DGAT1	Forward	TCAGATTGAGAAGCGCCTGG
	Reverse	ACGGAACCCACTGGAGTGAT
F4/80	Forward	AGTACGATGTGGGGCTTTTG
	Reverse	CCCCATCTGTACATCCCACT
FAS	Forward	CTGGACTCGCTCATGGGTG
	Reverse	CATTTCCTGAAGTTTCCGCAG
FGF21	Forward	CGAGGCTGAAAAGATGGCCT
	Reverse	GCGGCAGAAGAGAGCTATAACA
FGFR1	Forward	GTAGCTCCCTACTGGACATCC
	Reverse	GCATAGCGAACCTTGTAGCCTC
FOXP3	Forward	GAAACAGCACATTCCCAGAGTTC
	Reverse	ATGGCCCAGCGGATGAG
GAPDH	Forward	TGTGTCCGTCGTGGATCTGA
	Reverse	CCTGCTTCACCACCTTCTTGAT
IFN-γ	Forward	TCAAGTGGCATAGATGTGGAAGAA
	Reverse	TGGCTCTGCAGGATTTTCATG
IL-1β	Forward	AGACAGGTCGCTCAGGGTCA
	Reverse	AAGTGGTTGCCCATCAGAGG

IL-6	Forward	TCCAGTTGCCTTCTTGGGAC
	Reverse	AGTCTCCTCTCCGGACTTGT
β-klotho	Forward	GGTCTCCGGGGAATGAATGG
	Reverse	TAACAGCTCGCAGCAGAACA
ND5	Forward	AGCATTCGGAAGCATCTTTG
	Reverse	TTGTGAGGACTGGAATGCTG
Occludin	Forward	ATGTCCGGCCGATGCTCTC
	Reverse	TTTGGCTGCTCTTGGGTCTGTAT
PPARα	Forward	GTACGGTGTGTATGAAGCCATCTT
	Reverse	GCCGTACGCGATCAGCAT
PPARγ	Forward	AGTGGAGACCGCCCAGG
	Reverse	GCAGCAGGTTGTCTTGGATGT
PGC-1α	Forward	CCTGAAGCCGGGAGAGAATG
	Reverse	TAGCCAGCAGAGACTGTGGA
Prdm16	Forward	GAAGTCACAGGAGGACACGG
	Reverse	CTCGCTCCTCAACACACCTC
SIRT1	Forward	TTGGCACCGATCCTCGAAC
	Reverse	CCCAGCTCCAGTCAGAACTAT
SCD1	Forward	TCAACTTCACCACGTTCTTCA
	Reverse	CTCCCGTCTCCAGTTCTCTT
SREBP1c	Forward	AGCAGCCCCTAGAACAAACAC
	Reverse	CAGCAGTGAGTCTGCCTTGAT
TNF-α	Forward	GAGGCTCCAGTGAATTCGGA
	Reverse	CACAAGATGCTGGGACAGTGA
UCP1	Forward	ACTGCCACACCTCCAGTCATT
	Reverse	CTTTGCCTCACTCAGGATTGG
ZO-1	Forward	TTTTTGACAGGGGGAGTGG
	Reverse	TGCTGCAGAGGTCAAAGTTCAAG

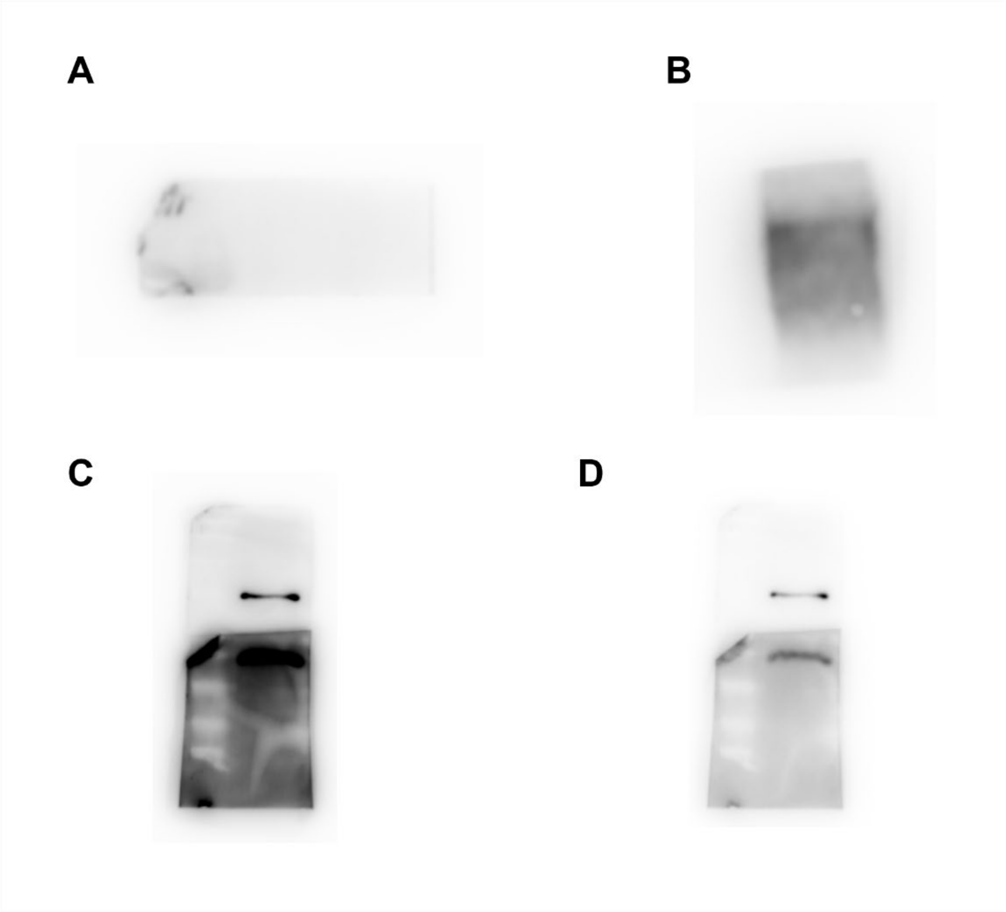

Supplementary Figure S1. Full length western blot of Exosome markers (Figure 1A). (A) Calnexin (B) CD63 (C) Syntenin (D) CD81.

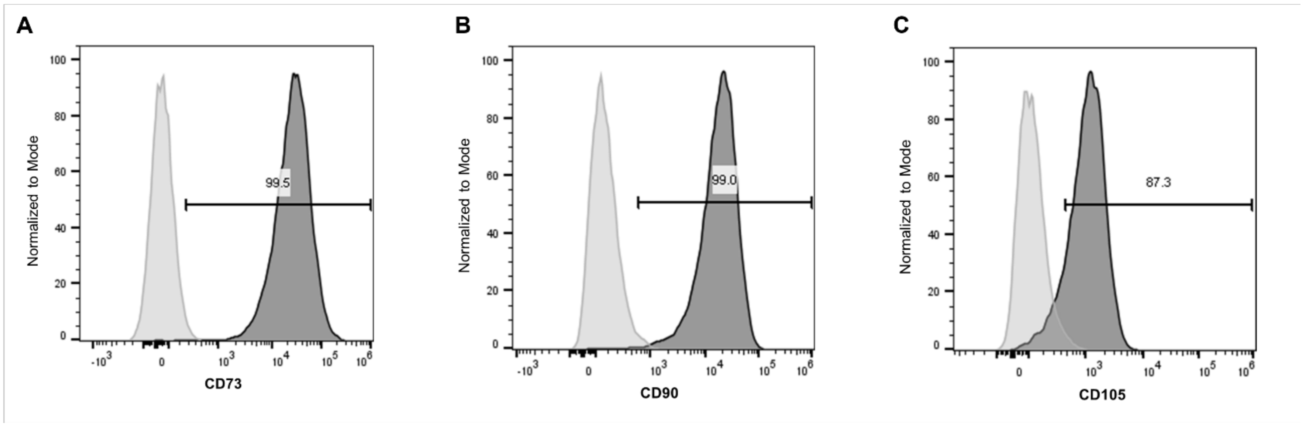

Supplementary Figure S2. Mesenchymal stem cell characterization. Flow cytometry analysis demonstrated that hWJMSCs were uniformly positive for expressions of (A) CD73, (B) CD90, and (C) CD105.
